# Low-molecular-weight heparins in atrial fibrillation: clinical scenarios, limitations, and future perspectives

**DOI:** 10.3389/fphar.2026.1853400

**Published:** 2026-10-07

**Authors:** Sabina Mędrek, Magdalena Zaborowska-Szmit, Sebastian Szmit

**Affiliations:** 1 Department of Cardio-Oncology, Centre of Postgraduate Medical Education, Warsaw, Poland; 2 Department of Cardiology and Cardio-Oncology Unit, Subcarpathian Oncological Center, Brzozow, Poland; 3 Department of Lung Cancer and Thoracic Tumours, Maria Skłodowska-Curie National Research Institute of Oncology, Warsaw, Poland; 4 Cardio-Oncology Unit, Maria Skłodowska-Curie National Research Institute of Oncology, Warsaw, Poland

**Keywords:** anticoagulation, atrial fibrillation, cancer, direct oral anticoagulant, low-molecular-weight heparin (LMWH), stroke prevention, thrombocytopenia

## Abstract

**Introduction:**

Atrial fibrillation (AF) is associated with a significantly increased risk of thromboembolic complications, including ischaemic stroke. Despite the dominant role of oral anticoagulants, low-molecular-weight heparins (LMWHs) are still used in selected clinical scenarios.

**Aim:**

The aim of this review was to summarise the current clinical role of LMWHs in atrial fibrillation, with particular emphasis on available evidence, practical limitations and selected complex clinical scenarios.

**Methods:**

A review based on the analysis of available clinical trials, guidelines, and pharmacological data.

**Results:**

Low-molecular-weight heparins are used primarily for bridging therapy and in situations where oral anticoagulation is temporarily contraindicated or not feasible. However, robust evidence supporting the long-term use of LMWHs for stroke prevention in atrial fibrillation remains limited.

**Conclusion:**

Low-molecular-weight heparins should not be considered standard chronic therapy for atrial fibrillation. Further studies are needed to better define optimal anticoagulation strategies in patients with atrial fibrillation, particularly in complex clinical settings such as active cancer and thrombocytopenia.

## Introduction

Atrial fibrillation (AF) is the most common cardiac arrhythmia in the adult population and represents a major global health concern (Van Gelder et al., 2024; Hindricks et al., 2021). The incidence of AF increases with both age and the rising prevalence of cardiovascular diseases (Lane et al., 2017; Karatela and Calkins, 2025). Cancer patients have a higher prevalence of AF than the general population, which ranges from approximately 2% to over 10%, depending on cancer type and stage (Yu et al., 2025; Saliba et al., 2018; Ay et al., 2022). The highest rates are observed among patients with lung and gastrointestinal malignancies, as well as in those with a history of thoracic surgery (Yun et al., 2021). AF and cancer may share common pathophysiological mechanisms, including inflammation, oxidative stress, and endothelial dysfunction, which increase the risk of thromboembolic events (Ay et al., 2022). AF is one of the leading risk factors for ischaemic stroke, increasing its risk several-fold (Tracz et al., 2023; Yang et al., 2022). AF-related strokes account for a significant proportion of all ischaemic strokes and are characterised by higher mortality and poorer functional outcomes (Tracz et al., 2023; Tu et al., 2015). The pathophysiology of thrombus formation in AF is complex, encompassing haemodynamic disturbances, activation of the coagulation system, and endothelial dysfunction (Watson et al., 2009). Blood stasis in the left atrium, particularly in the left atrial appendage, promotes thrombus formation and may give rise to thromboembolic complications (Goldman et al., 1999; Cimmino et al., 2021). Antithrombotic prophylaxis therefore constitutes a key element in the management of AF patients.

Current clinical guidelines recommend oral anticoagulation for most AF patients who are at an increased risk of stroke, as assessed by the CHA_2_DS_2_-VA (Van Gelder et al., 2024), formerly CHA_2_DS_2_-VASc score (Hindricks et al., 2021). For many years, vitamin K antagonists, primarily warfarin, constituted the mainstay of anticoagulation therapy and remained the principal modality of oral anticoagulation for over half a century (Steinberg and Piccini, 2014; Zirlik and Bode, 2017). Clinical trials have shown that warfarin effectively reduces the risk of stroke and systemic embolism in AF patients (Hart et al., 2007). In recent years, direct oral anticoagulants (DOACs) have assumed a significantly greater role in anticoagulation therapy, demonstrating efficacy comparable to that of vitamin K antagonists and a more favourable safety profile (Van Gelder et al., 2024).

Despite clearly defined therapeutic recommendations favouring DOACs, LMWHs remain in clinical use for patients with AF, particularly in special populations such as cancer patients (Boriani et al., 2021; Nardi et al., 2024). Available scientific data indicate that the efficacy of LMWHs in stroke prevention in AF patients has not been clearly established, and its use may be associated with an increased risk of bleeding complications (Sorigue and Miljkovic, 2019). The aim of this review was to summarise the current clinical role of LMWHs in atrial fibrillation, with particular emphasis on available evidence, practical limitations, and selected complex clinical scenarios.

## Literature search strategy

This article was prepared as a narrative review. A literature search was performed using PubMed/MEDLINE and Google Scholar databases up to March 2026. The following keywords and combinations were used: “atrial fibrillation”, “low-molecular-weight heparin”, “LMWH”, “bridging therapy”, “cancer”, “stroke prevention”, and “anticoagulation”. Priority was given to randomized clinical trials, international guidelines, meta-analyses, and studies specifically addressing atrial fibrillation and cancer-associated thrombosis. Additional references were identified from cited bibliographies of relevant publications. Due to the limited number of randomized studies evaluating LMWHs in atrial fibrillation, selected observational studies and pharmacological analyses were also included.

## Pathophysiology of AF-related thrombosis

Thrombus formation in AF arises from the interaction of Virchow’s triad (Regazzoli et al., 2015), with the first component comprising atrial blood stasis due to uncoordinated electrical and mechanical atrial activity (Darlington and McCauley, 2020). Endothelial damage or dysfunction, which activates the coagulation cascade, is the second element (Hagendorff et al., 2025). Increased activity of the coagulation system, inflammation and cellular cross-talk within the atrial microenvironment contribute to thrombus formation in AF (Qureshi et al., 2023). In most cases, the thromboembolic material causing cerebral complications in nonvalvular AF originates from the left atrial appendage (Regazzoli et al., 2015; Zhan et al., 2018). Ischaemic stroke is the most common clinical manifestation of thromboembolic complications in AF (Pisters et al., 2012). In older, classical observations, the annual stroke risk in patients with AF was estimated at approximately 5%–7%; large contemporary population analyses report an annual incidence of ischaemic stroke of approximately 2.0% (Savelieva and Camm, 2000). Extracranial systemic embolic events (SEEs) are significantly less common than stroke and account for approximately 11.5% of all clinically recognized AF-related thromboembolic events, with cerebrovascular events representing most cases (Bekwelem et al., 2015). Acute limb ischaemia, most commonly affecting the lower limb and less frequently the upper limb—is the predominant clinical manifestation of peripheral embolism in AF; mesenteric, renal, and splenic embolism is also possible (Bekwelem et al., 2015; Gossage et al., 2006).

## The mechanism of action of LMWHs

LMWHs are a heterogeneous group of polysaccharides formed by controlled depolymerization of unfractionated heparin, resulting in their lower molecular weight and different pharmacological profile (Hirsh et al., 2001; Fareed et al., 2003). Their anticoagulant effect primarily enhances the activity of antithrombin, a key inhibitor of coagulation proteases, leading to the inhibition of factor Xa and, to a lesser extent, thrombin (factor IIa) (Hirsh et al., 2001; Fareed et al., 2003; Rezaie and Giri, 2020). LMWHs preferentially inhibit factor Xa due to the shorter polysaccharide chains, which are insufficient to simultaneously bind antithrombin and thrombin, thereby limiting their ability to inactivate factor IIa (Rezaie and Giri, 2020; Wolberg and Campbell, 2008). However, individual LMWHs differ significantly in pharmacodynamic properties, including the anti-Xa to anti-IIa activity ratio, which depends on the depolymerization method and average molecular weight, typically ranging from about 1.5:1 to 4:1 (Eritsland, 2005; Samama and Gerotziafas, 2000). For instance, enoxaparin demonstrates significantly greater anti-factor Xa activity compared to dalteparin, achieving up to 30%–100% stronger inhibition of factor Xa, whereas tinzaparin is characterised by a relatively greater effect on thrombin (Miano et al., 2018). Differences between LMWHs also encompass their effects on other components of the coagulation system, including the ability to release tissue factor pathway inhibitor (TFPI), which further modulates thrombin generation and the prothrombotic response (Hirsh et al., 2001). Inhibition of factor Xa leads to a reduction in thrombin formation, which plays a central role in the conversion of fibrinogen to fibrin and thrombus stabilisation; therefore, the dominant anti-Xa effect of LMWH effectively limits thrombus formation (Krishnan et al., 2004). Compared to unfractionated heparin, LMWHs are characterised by a more predictable pharmacokinetic profile, higher bioavailability after subcutaneous administration, and a longer half-life (Huang and Shimamura, 1998). These properties enable the use of LMWHs at fixed doses without routine monitoring of laboratory coagulation parameters in most patients (Turpie, 1998). Due to their favourable safety and efficacy profile, LMWHs have been widely used in the prevention and treatment of venous thromboembolism (Lyman, 2009). Although LMWHs demonstrate effective anticoagulant activity, their role in long-term stroke prevention in atrial fibrillation remains limited due to the lack of robust randomized evidence supporting chronic use in this setting.

## Clinical scenarios of LMWH use in AF patients

LMWHs constitute an important component of anticoagulant therapy in selected AF clinical cases, particularly during temporary modifications of oral therapy, periods of increased thromboembolic risk, or when oral anticoagulants are contraindicated.

Bridging therapy during temporary discontinuation of oral anticoagulants (particularly vitamin K antagonists such as warfarin), for example, before planned surgical or invasive procedures, is one of the main indications for LMWH use in AF patients (Ayoub et al., 2016). LMWHs are administered to maintain continuous anticoagulation and minimize the risk of stroke or systemic embolism. However, current guidelines do not recommend routine heparin bridging in most AF patients as this strategy increases the risk of perioperative bleeding without significantly reducing the risk of arterial thromboembolism. In the BRIDGE trial involving 1884 AF patients requiring temporary interruption of warfarin therapy, arterial thromboembolism occurred in 0.4% of patients without bridging and 0.3% with dalteparin bridging, whereas major bleeding occurred in 1.3% and 3.2%, respectively (Douketis et al., 2015). Particular caution is warranted as patients at highest stroke risk were underrepresented in randomised trials; thus, decisions regarding bridging therapy in this group should be based on an individual assessment of the balance between thromboembolic and haemorrhagic risk (Garwood et al., 2017; Siddiqui et al., 2021). Bridging therapy may still be considered in selected patients at very high thromboembolic risk, including those with recent stroke or transient ischemic attack, mechanical heart valves or very high thromboembolic risk as assessed by contemporary risk stratification tools (Douketis et al., 2015). LMWH bridging is generally not recommended in patients treated with DOACs as the short and predictable periods of withdrawal of these agents before a procedure usually allow it to be performed safely without additional heparin. In the PAUSE trial, a standardised strategy of temporary DOAC discontinuation without heparin bridging was associated with a low incidence of major bleeding and arterial thromboembolic events in AF patients receiving apixaban, dabigatran, or rivaroxaban (Douketis et al., 2019). Furthermore, there is no need to interrupt oral anticoagulation and, therefore, no need for heparin bridging for many minor procedures associated with a low risk of bleeding (Douketis et al., 2015).

LMWHs may be used as an alternative in patients with contraindications to oral anticoagulants, such as significant drug interactions, malabsorption, or difficulty maintaining a stable INR, and may be used both temporarily and, in some cases, chronically (Boriani et al., 2021; Farmakis et al., 2022).

LMWHs are commonly utilised as a temporary anticoagulant strategy in the hospital setting, particularly in patients with acute comorbidities (such as infections, worsening heart failure, or immobilisation). Their use is particularly justified in situations requiring a rapid anticoagulant effect and predictable pharmacokinetics (Polania Gutierrez and Rocuts, 2026).

LMWHs may also be used temporarily at the onset of anticoagulant therapy before the full therapeutic effect of oral anticoagulants is achieved (e.g., when initiating therapy with vitamin K antagonists) (Kahlon et al., 2016; Witt et al., 2016).

## Scientific evidence for the efficacy of LMWHs in stroke prevention in patients with AF

Oral anticoagulants, whose effectiveness in reducing the risk of thromboembolic complications has been clearly demonstrated across many clinical trials, remain the mainstay of stroke prevention in patients with AF (Steinberg and Piccini, 2014). Unlike oral anticoagulants, the role of LMWHs in long-term stroke prevention in AF patients has not been clearly established in randomised trials (Pappas et al., 2017).

Heparin in Acute Embolic Stroke Trial (HAEST), which compared dalteparin vs. aspirin in patients with acute ischaemic stroke (AIS) and AF, is one of the key studies in this area. HAEST showed no significant superiority of LMWH over aspirin in reducing stroke recurrence or death (Berge et al., 2000).

Similar findings were reported in the TAIST study, which compared tinzaparin vs. aspirin in patients with AIS. This study showed no significant differences in clinical outcomes between the analysed therapeutic strategies (Bath et al., 2001).

Previous studies, such as FISS, evaluating the efficacy of LMWHs in patients with AIS demonstrated no significant advantage of this therapy over standard treatment in improving neurological outcomes (Kay et al., 1995).

Studies comparing LMWHs with unfractionated heparin showed no significant differences in primary endpoints such as recurrent stroke, myocardial infarction, or mortality (Feiz et al., 2016). Meta-analyses indicate that the use of LMWHs in AIS does not significantly reduce the risk of recurrent stroke but is associated with an increased risk of bleeding complications (Sandercock et al., 2008).

Studies evaluating bridging therapy, including BRIDGE, have shown no significant reduction in the risk of thromboembolic complications with LMWH in AF patients, while noting a concomitant increase in the risk of bleeding complications (Douketis et al., 2015).

Based on available clinical data, LMWHs have not been shown to be effective for routine, long-term stroke prevention in patients with AF; therefore, their use in this indication remains limited to selected clinical scenarios (Doherty et al., 2017). It should be emphasised that the available data are limited by the absence of large, randomised clinical trials evaluating LMWH use in AF patients, potentially compromising the strength of the conclusions drawn. The key clinical studies evaluating the efficacy and safety of LMWHs in patients with atrial fibrillation and/or acute ischaemic stroke are summarised in Table 1.

**TABLE 1:** Key clinical studies evaluating the efficacy and safety of low-molecular-weight heparins (LMWHs) in patients with atrial fibrillation and/or acute ischaemic stroke

Study (reference)	Population	Sample size (n)	Design	Intervention and comparator	Treatment duration / follow-up	Primary efficacy outcome	Key safety outcome(s)	Main conclusion
HAEST (Berge et al., 2000)	Atrial fibrillation with acute ischaemic stroke; treatment started within 30 h of stroke onset	449	Multicentre, randomised, double-blind, double-dummy trial	Dalteparin 100 anti-Xa IU/kg SC twice daily vs aspirin 160 mg once daily	14 days; follow-up to 3 months	Recurrent ischaemic stroke within 14 days: 8.5% with dalteparin vs 7.5% with aspirin (OR 1.13, 95% CI 0.57–2.24)	Symptomatic cerebral haemorrhage: 2.7% vs 1.8%. Symptomatic or asymptomatic cerebral haemorrhage: 11.6% vs 14.2%. No significant between-group difference.	Dalteparin was not superior to aspirin for preventing early recurrent stroke and did not improve functional outcome or mortality.
TAIST (Bath et al., 2001)	Acute ischaemic stroke, with or without atrial fibrillation; treatment started within 48 h	1,486 randomised (1,484 treated)	Randomised, double-blind, aspirin-controlled trial	Tinzaparin 175 anti-Xa IU/kg once daily (high dose) or 100 anti-Xa IU/kg once daily (medium dose) vs aspirin 300 mg once daily	Up to 10 days; follow-up to 6 months	Independence at 6 months (mRS 0–2): 41.5% with high-dose tinzaparin, 42.4% with medium-dose tinzaparin, and 42.5% with aspirin	Symptomatic intracerebral haemorrhage occurred in 7 patients receiving high-dose tinzaparin vs 1 receiving aspirin. High-dose tinzaparin reduced symptomatic DVT (0 vs 9 cases with aspirin).	Neither tinzaparin dose improved functional outcome vs aspirin; high-dose tinzaparin increased symptomatic intracranial haemorrhage.
FISS (Kay et al., 1995)	Acute ischaemic stroke; treatment started within 48 h of symptom onset	312	Randomised, double-blind, placebo-controlled trial	Nadroparin 4,100 anti-factor Xa IU SC twice daily (high dose) or once daily (low dose) vs placebo	10 days; follow-up to 6 months	Death or dependency at 6 months: 45% with high-dose nadroparin, 52% with low-dose nadroparin, and 65% with placebo (P = 0.005 for trend)	No significant differences among groups in death, haemorrhagic transformation, or other complications during the 10-day treatment period	The trial reported a dose-dependent improvement in death or dependency at 6 months, without a significant increase in short-term complications.
BRIDGE (Douketis et al., 2015)	Atrial fibrillation requiring temporary interruption of warfarin for an elective operation or invasive procedure	1,884	Randomised, double-blind, placebo-controlled trial	Dalteparin 100 IU/kg SC twice daily vs matching placebo (no bridging)	From 3 days before the procedure until 24 h before it, then for 5–10 days after the procedure; follow-up 30 days	Arterial thromboembolism: 0.4% without bridging vs 0.3% with dalteparin (P = 0.01 for non-inferiority)	Major bleeding: 1.3% without bridging vs 3.2% with dalteparin (P = 0.005). Minor bleeding: 12.0% vs 20.9% (P < 0.001).	Forgoing bridging was non-inferior for arterial thromboembolism and reduced major and minor bleeding.

Abbreviations: AF, atrial fibrillation; CI, confidence interval; DVT, deep vein thrombosis; LMWH, low-molecular-weight heparin; mRS, modified Rankin Scale; OR, odds ratio; SC, subcutaneous.

## LMWHs in cancer patients with AF

AF and cancer often coexist, complicating the therapeutic process due to overlapping prothrombotic mechanisms and an increased risk of bleeding (Boriani et al., 2021). It is estimated that approximately 2%–10% of cancer patients are diagnosed with AF, with this frequency potentially higher in certain patient groups and clinical cases (Sorigue and Miljkovic, 2019). At the same time, cancer patients are at an increased risk of both thromboembolic complications and bleeding, which significantly complicates the optimal selection of anticoagulant therapy (Sorigue and Miljkovic, 2019). Assessing the risk of thromboembolism and bleeding in this population poses a challenge as commonly used risk scores, including CHA_2_DS_2_-VA/CHA_2_DS_2_-VASc and HAS-BLED, have not been fully validated in cancer patients (Boriani et al., 2021).

Significant heterogeneity in therapeutic management and a lack of clear treatment regimens in this population translate into suboptimal use of anticoagulation (Boriani et al., 2021). The 2022 ESC guidelines on cardio-oncology recommend an individualized approach based on the assessment of thrombotic risk, bleeding risk, potential drug-drug interactions, and patient-related factors (TBIP framework), highlighting the complexity of anticoagulation management in patients with cancer and atrial fibrillation (Lyon et al., 2022). Available data indicate that a considerable proportion of cancer patients with AF receive inappropriate or subtherapeutic anticoagulation, partly reflecting uncertainty regarding the optimal anticoagulation strategy in this population (Nardi et al., 2024). Importantly, much of the available evidence derives from studies on cancer-associated venous thromboembolism rather than atrial fibrillation-specific populations; therefore, extrapolation of these findings to AF management should be interpreted cautiously (Sorigue, 2020). Their use in this group of patients is mainly limited to clinical scenarios where oral anticoagulants are difficult to use or contraindicated (Figure 1) (Boriani et al., 2021). In observational analyses, LMWH use in cancer patients with AF was associated with higher rates of stroke and mortality compared with vitamin K antagonists, although interpretation is limited by substantial selection bias and heterogeneity of treated populations (Chai-Adisaksopha et al., 2023). A possible explanation for these observations is the use of LMWHs at suboptimal doses or for a limited duration, potentially resulting in inadequate protection against thromboembolic complications (Nardi et al., 2024). Furthermore, the absence of standardised LMWH dosing regimens in AF, along with the limited data from randomised trials, substantially restricts the ability to evaluate their efficacy in this population (Sorigue, 2020).

**FIGURE 1:**
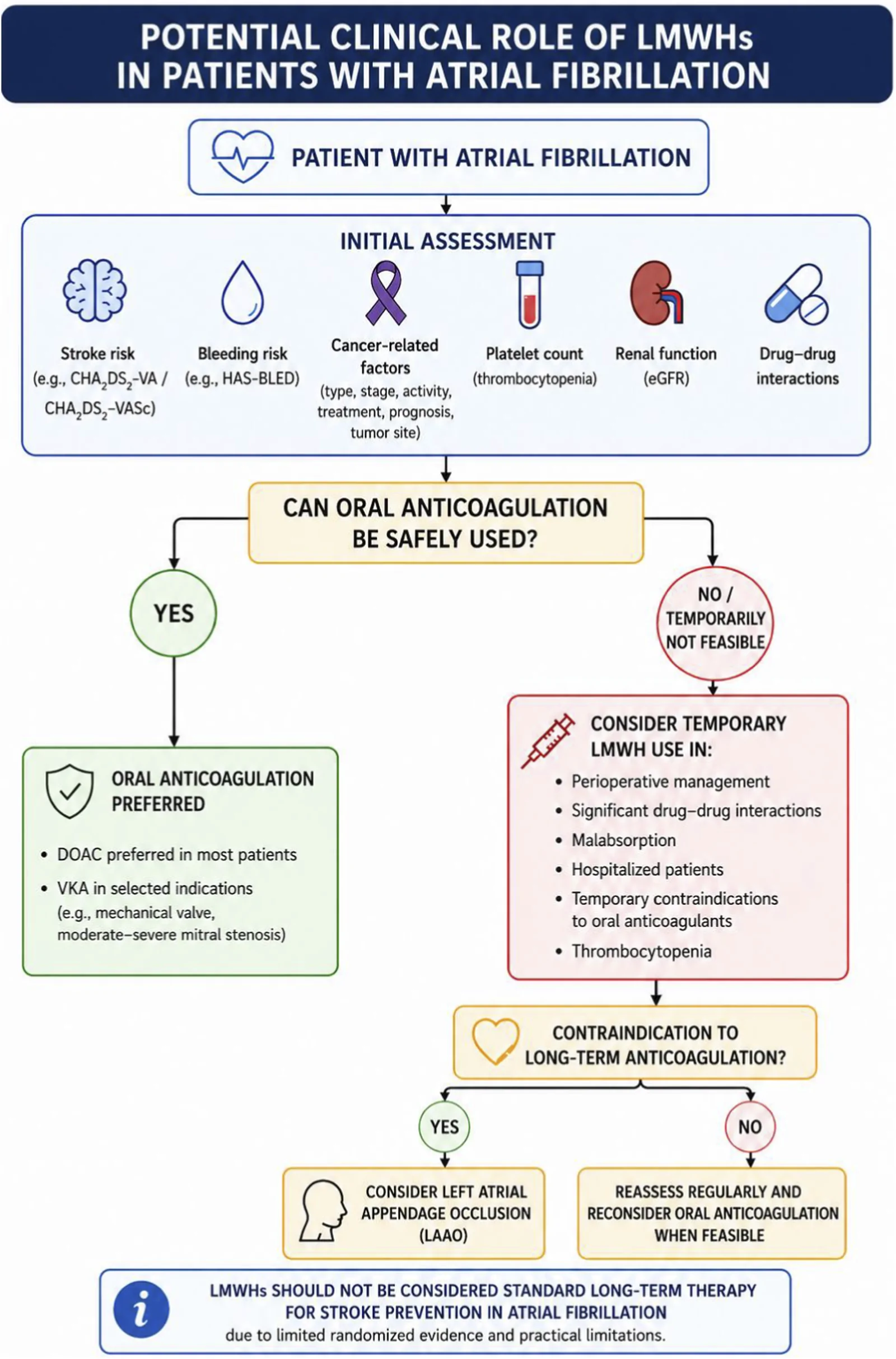
Potential clinical role of low-molecular-weight heparins (LMWHs) in patients with atrial fibrillation. Legend: Proposed algorithm illustrating clinical situations in which LMWHs may be considered in patients with atrial fibrillation when oral anticoagulation is temporarily contraindicated, not feasible, or associated with significant limitations. Assessment should include thromboembolic risk (CHA_2_DS_2_-VA or CHA_2_DS_2_-VASc), bleeding risk, cancer-related factors, platelet count, renal function, and potential drug–drug interactions. LMWHs may be used as a temporary alternative in selected clinical scenarios; however, they should not be considered standard long-term therapy for stroke prevention in atrial fibrillation because of limited randomized evidence supporting their chronic use. Abbreviations: AF, atrial fibrillation; CHA_2_DS_2_-VA, Congestive heart failure, Hypertension, Age ≥75 years (2 points), Diabetes mellitus, prior Stroke/transient ischemic attack/systemic embolism (2 points), Vascular disease, Age 65–74 years; CHA_2_DS_2_-VASc, Congestive heart failure, Hypertension, Age ≥75 years (2 points), Diabetes mellitus, prior Stroke/transient ischemic attack/systemic embolism (2 points), Vascular disease, Age 65–74 years, Sex category (female); DOAC, direct oral anticoagulant; eGFR, estimated glomerular filtration rate; LAAO, left atrial appendage occlusion; LMWH, low-molecular-weight heparin; VKA, vitamin K antagonist.

In recent years, DOACs have gained increasing importance in the treatment of patients with AF and cancer, with available analyses demonstrating at least comparable, and in some cases superior, efficacy and safety profiles compared with vitamin K antagonists (Shah et al., 2018). One of the practical benefits of LMWHs in cancer patients with AF is their relatively low potential for drug–drug interactions compared with oral anticoagulants, particularly in those receiving combination anticancer therapy. Therefore, LMWHs may be the preferred therapeutic option in situations where the use of oral anticoagulants involves a high risk of pharmacological interactions (Wang, 2022).

Thrombocytopenia, which is associated with both the disease itself and its treatment, is a significant factor limiting the use of anticoagulation in cancer patients. Thrombocytopenia increases the risk of bleeding without eliminating the risk of thrombosis (Szmit et al., 2025). Therapeutic decisions depend on the severity of thrombocytopenia, with dose tapering or temporary discontinuation of anticoagulation commonly employed in clinical practice when the platelet count falls below 50,000/µL (Falanga et al., 2022). According to EHA recommendations, full-dose anticoagulation is generally acceptable when platelet count exceeds 50,000/µL, whereas dose reduction or temporary interruption may be considered in more severe thrombocytopenia depending on thrombotic and bleeding risk (Figure 2). In the context of AF, this poses a particular clinical challenge, as interruption or reduction of anticoagulant dosing may afford insufficient protection against stroke (Iyengar et al., 2023). In clinical practice, agents with shorter half-lives or those permitting flexible dose modification are often preferred to enable easier treatment adjustments (Patell and Zwicker, 2022). Patients with a history of bleeding or those with cancers associated with high bleeding risk, particularly in the gastrointestinal or genitourinary tract, present an additional challenge (Chai-Adisaksopha et al., 2023). These patients face an increased risk of recurrent bleeding, which influences decisions on anticoagulant initiation or continuation (Escobar et al., 2022). Treatment decisions thus necessitate an individualised assessment of the balance between thrombotic and bleeding risks (Sanz and Gómez, 2019).

**FIGURE 2:**
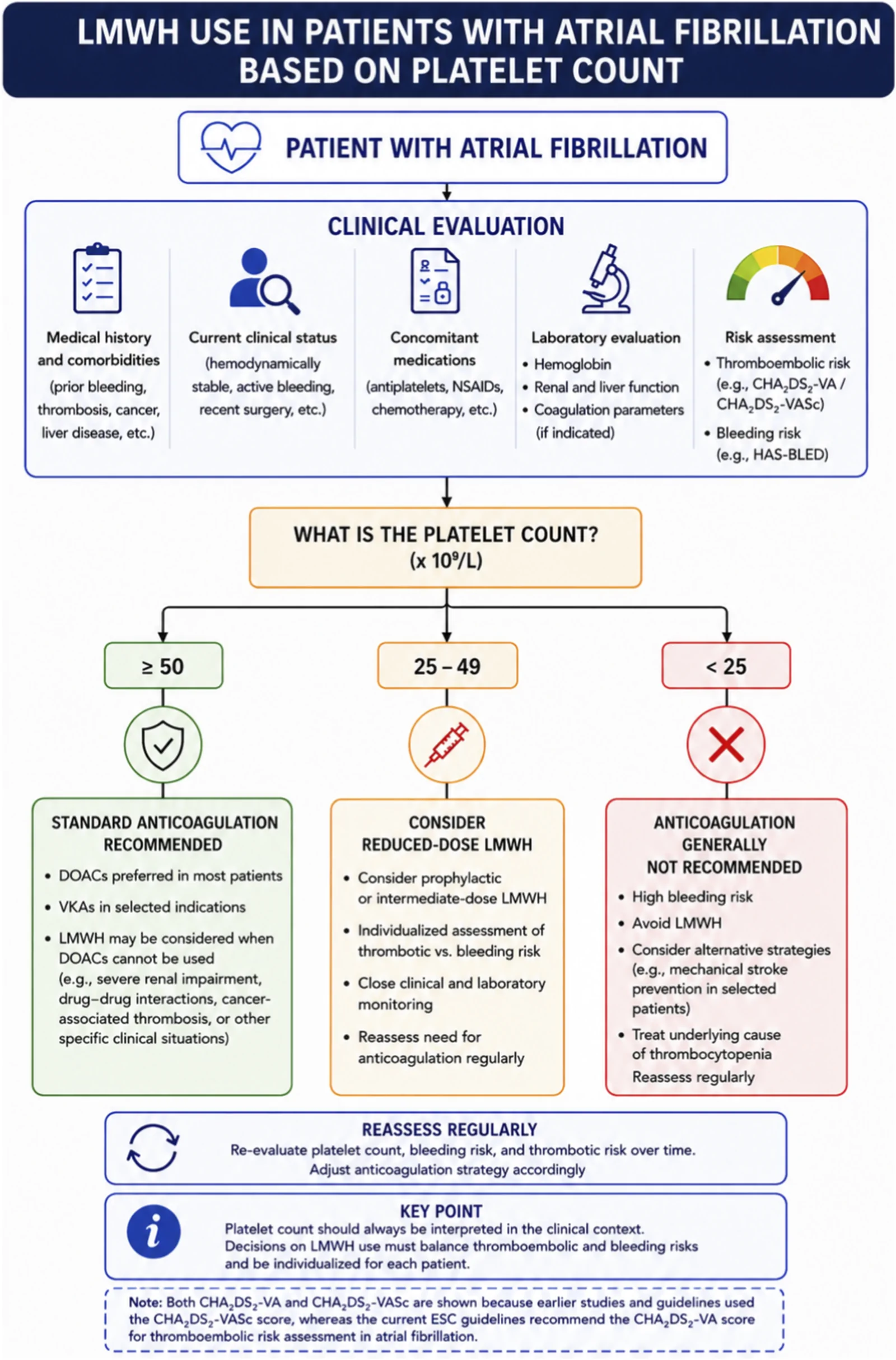
Proposed algorithm for anticoagulation management in patients with atrial fibrillation and thrombocytopenia based on platelet count thresholds. Legend: Proposed approach to anticoagulation management in patients with atrial fibrillation and thrombocytopenia according to platelet count. In patients with platelet counts ≥50 × 10^9^/L, standard anticoagulation is generally preferred, with direct oral anticoagulants (DOACs) representing the first-line option in most cases. Low-molecular-weight heparins (LMWHs) may be considered when DOACs cannot be used because of specific clinical circumstances, such as significant drug–drug interactions, malabsorption, severe renal impairment, or temporary contraindications to oral anticoagulation. In patients with platelet counts of 25–49 × 10^9^/L, reduced-dose LMWH may be considered following individualized assessment of thromboembolic and bleeding risks. For platelet counts <25 × 10^9^/L, anticoagulation is generally not recommended because of the substantially increased risk of bleeding. Clinical decisions should be individualized and reassessed regularly according to changes in platelet count, bleeding risk, thromboembolic risk, and the underlying cause of thrombocytopenia. Abbreviations: AF, atrial fibrillation; CHA_2_DS_2_-VA, Congestive heart failure, Hypertension, Age ≥75 years (2 points), Diabetes mellitus, prior Stroke/transient ischemic attack/systemic embolism (2 points), Vascular disease, Age 65–74 years; CHA_2_DS_2_-VASc, Congestive heart failure, Hypertension, Age ≥75 years (2 points), Diabetes mellitus, prior Stroke/transient ischemic attack/systemic embolism (2 points), Vascular disease, Age 65–74 years, Sex category (female); DOAC, direct oral anticoagulant; HAS-BLED, Hypertension, Abnormal renal/liver function, Stroke, Bleeding history or predisposition, Labile INR, Elderly, Drugs/alcohol; LMWH, low-molecular-weight heparin; NSAID, nonsteroidal anti-inflammatory drug; VKA, vitamin K antagonist.

Renal dysfunction and frailty may further complicate anticoagulation management in patients with AF and cancer, as both conditions are associated with increased risks of bleeding, thromboembolic events, altered drug pharmacokinetics, and reduced treatment tolerance. Chronic kidney disease is present in approximately 30%–40% of patients with atrial fibrillation and is independently associated with adverse cardiovascular outcomes. The risk becomes particularly relevant in patients with moderate-to-severe renal impairment (eGFR <60 mL/min/1.73 m^2^), while severe renal dysfunction (eGFR <30 mL/min/1.73 m^2^) may substantially limit anticoagulation options, increase the risk of drug accumulation and bleeding, and require dose adjustment or alternative treatment strategies according to current guideline recommendations (Van Gelder et al., 2024; Fanola et al., 2018). Frailty is increasingly recognized as an important determinant of outcomes in patients with AF. Depending on the population studied and the assessment method used, frailty affects approximately 20%–40% of older patients with atrial fibrillation. Frail patients are characterized by a higher burden of comorbidities, polypharmacy, functional impairment, falls, hospitalization, and mortality. Several validated instruments, including the Clinical Frailty Scale (CFS), Fried Frailty Phenotype, and Frailty Index, may be used to assess frailty and support individualized therapeutic decision-making. Although frailty alone should not be considered a contraindication to anticoagulation, careful assessment of net clinical benefit and close follow-up are warranted in this vulnerable population (Wilkinson et al., 2019). These factors may substantially influence therapeutic decision-making and therefore require careful individualisation of anticoagulant therapy and close clinical monitoring.

In selected patients with contraindications to long-term anticoagulation, left atrial appendage occlusion may represent an alternative stroke prevention strategy, although evidence in active cancer populations remains limited. Current evidence supporting LAAO in patients with active cancer is derived primarily from observational studies; therefore, careful patient selection and individualized assessment of procedural benefits and risks are required (Cimmino et al., 2021).

Interpretation of observational data in cancer populations warrants caution, as treatment selection, often influenced by clinical complexity, may introduce selection bias and limit result generalisability.

## Potential adverse reactions of LMWHs

Bleeding, which may range in severity from mild to life-threatening, is the most important complication of LMWH anticoagulant therapy (Costantino et al., 2012; Bikdeli et al., 2022). Major bleeding represents the most clinically significant complication of LMWH therapy and occurs more frequently in elderly patients, cancer populations and those with renal dysfunction (Costantino et al., 2012; Bikdeli et al., 2022; Angelini et al., 2019).

Heparin-induced thrombocytopenia (HIT), a serious, immune-mediated adverse effect, is another significant complication. Its mechanism involves the formation of antibodies against platelet factor 4 (PF4)–heparin complexes, leading to platelet activation and consumption and, paradoxically, an increased risk of thromboembolic complications. HIT is typically characterised by a substantial decrease in platelet count of ≥50% from baseline, most commonly occurring between days 5 and 10 of therapy, and may result in serious complications, including venous and arterial thrombosis. Although the risk of HIT is lower with LMWHs than with unfractionated heparin, this complication remains clinically important because of its potential thrombotic consequences (Warkentin, 2003; May and Cuker, 2024).

The requirement for daily subcutaneous LMWH administration constitutes a major limitation of this therapy, as injection-site discomfort and associated adverse effects may compromise patient adherence (Horzum et al., 2026; Hutchinson et al., 2019).

## Limitations of scientific evidence in the context of persistent LMWH use in patients with AF

Despite the limited availability of high-quality evidence supporting the long-term use of LMWHs in patients with AF, these agents are still employed in clinical practice (Steffel et al., 2021), (Hindricks et al., 2021). One contributing factor may be the longstanding clinical experience with LMWHs in venous thromboembolism and cancer-associated thrombosis, despite the limited AF-specific evidence base (Kearon et al., 2016; Liu et al., 2025). Furthermore, LMWHs were for many years the standard first-line therapy for cancer-associated thrombosis and therefore have an established therapeutic role in this patient population (Lee et al., 2003; Khorana et al., 2018).

The management of patients with cancer and AF is particularly complex from a clinical standpoint. This complexity arises from the coexistence of an elevated risk of both thromboembolic and haemorrhagic complications, as well as many potential drug–drug interactions, which may prompt the selection of therapies with a well-established safety profile (López-Fernández et al., 2019). Concerns regarding potential pharmacokinetic and pharmacodynamic interactions between DOACs and ongoing anticancer therapy constitute an important factor influencing therapeutic decision-making (Farmakis et al., 2022; Steffel et al., 2021). Furthermore, the limited availability of large, randomised clinical trials in this specific patient population leads to substantial heterogeneity in clinical practice and necessitates the individualisation of treatment strategies (Farmakis et al., 2022; Zhu et al., 2018). Therefore, current use of LMWHs in atrial fibrillation is driven largely by clinical necessity and expert judgement rather than by robust randomized evidence.

## Pharmacological implications and future directions for the development of new anticoagulants

The limited efficacy of LMWHs in long-term stroke prevention in patients with AF highlights important gaps in current therapeutic strategies. Despite their anticoagulant properties, robust evidence supporting the routine use of LMWHs for stroke prevention in atrial fibrillation remains limited (Pappas et al., 2017; Doherty et al., 2017; Steffel et al., 2021; Hindricks et al., 2021).

DOACs have demonstrated favourable efficacy and safety profiles in randomized clinical trials and currently represent the preferred anticoagulation strategy for most patients with atrial fibrillation (Carnicelli et al., 2022). Future directions in the development of antithrombotic pharmacotherapy may encompass selective inhibition of factors XI and XII—promising targets owing to their prominent role in thrombosis and limited involvement in physiological haemostasis—as well as strategies to achieve more selective and safer mechanisms of action for antithrombotic agents (Bernardi et al., 2026; Capodanno et al., 2025). Future research should focus on prospective studies evaluating anticoagulation strategies specifically in patients with atrial fibrillation and active cancer, as this population remains underrepresented in randomized clinical trials. Such studies are needed to better define the role of LMWHs and other anticoagulant strategies in complex clinical settings characterized by a high risk of both thrombosis and bleeding. A more comprehensive understanding of the complex pathophysiological mechanisms underlying thrombosis in AF could facilitate the development of more effective and safer antithrombotic therapies (Dong et al., 2026). These approaches are consistent with an emerging paradigm in antithrombotic therapy that seeks to uncouple the processes of thrombogenesis and haemostasis (Bernardi et al., 2026).

## Conclusion

- LMWHs may be considered a temporary alternative in selected situations where oral anticoagulation is not feasible, including severe drug–drug interactions, malabsorption, perioperative management, or temporary contraindications to oral agents
- LMWHs should not be regarded as a standard strategy for long-term stroke prevention in patients with atrial fibrillation
- In cancer patients, therapeutic decision-making should be guided by assessments of potential drug–drug interactions, bleeding risk, and the overall clinical context
- The coexistence of cancer, renal dysfunction, and frailty represents a particularly challenging clinical scenario, as all three conditions simultaneously increase both thrombotic and bleeding risk while limiting available anticoagulation options.
- Current evidence does not support routine long-term use of LMWHs for stroke prevention in atrial fibrillation outside selected clinical scenarios.
